# A Stepped Care Approach to Promoting Engagement in a Digital Health Intervention: Secondary Analysis of a Randomized Controlled Trial

**DOI:** 10.2196/80921

**Published:** 2026-05-04

**Authors:** Esha Dwibedi, Wen You, Lee M Ritterband, Donna-Jean P Brock, Annie L Reid, Christina Frederick, Jamie M Zoellner

**Affiliations:** 1 Department of Public Health Sciences School of Medicine University of Virginia Christiansburg, VA United States; 2 Department of Public Health Sciences School of Medicine University of Virginia Charlottesville, VA United States; 3 Department of Psychiatry and Neurobehavioral Sciences School of Medicine University of Virginia Charlottesville, VA United States

**Keywords:** digital health interventions, engagement, stepped care, implementation cost, simulations

## Abstract

**Background:**

Digital health interventions (DHIs) often struggle with participant engagement. A stepped care approach, starting with low-resource intensity strategies and escalating as needed, can optimize resource use. Yet its application and cost implications remain underexplored.

**Objective:**

This study uses data from the iSIPsmarter experimental arm of a 2-group randomized controlled trial targeting sugar-sweetened beverage consumption in rural Appalachia. This study examines the demand and implementation costs associated with iSIPsmarter’s stepped care engagement approach and simulates how variations in monitoring efficiency, demand, and stepped care intensity influence resource use and implementation costs to inform future implementation.

**Methods:**

iSIPsmarter’s stepped care process combined automated and human-supported components to enhance engagement across 6 web-based modules (“Cores”) over 9 weeks. Participants who did not complete a Core received an automated email, followed by stepped care if still incomplete: a text (step 1, low-resource intensity) after 7 days and up to 3 telephone attempts (step 2, high-resource intensity) after another 7 days. Staff time was tracked to estimate implementation costs: monitoring averaged 3 minutes (US $1.68), texts 2.83 minutes (US $1.58), and calls 5.1 minutes (US $2.85). Simulations explored 18 scenarios varying monitoring efficiency (20%, 50%, and 80% of trial-observed monitoring time and costs), stepped care demand (20%, 50%, and 80% of participants needing stepped care), and intervention intensity (low vs high).

**Results:**

Among 126 participants, the mean stepped care contact was 1.2 (SD 1.3): 52 (41%) required none, 42 (33%) required 1 Core contact, 26 (21%) required 2, and 7 (6%) required 3. On average, participants completed 5.2 (SD 1.6) of 6 Cores. The mean stepped care implementation time per participant was 26.46 (SD 11.02) minutes, with a corresponding mean cost of US $14.80 (SD 6.16). Monitoring accounted for 78% of total cost (mean cost US $11.61, SD 2.37), with initial monitoring contributing 58% of total cost (mean cost US $8.51, SD 2.35). Simulations showed variation in time and cost based on monitoring efficiency. In low-demand, low-intensity scenarios, efficient monitoring required mean of 7.47 (95% CI 7.36-7.57) minutes and mean cost of US $4.18 (95% CI 4.12-4.24), while inefficient monitoring required a mean of 19.58 (95% CI 19.21-19.95) minutes and mean cost of US $10.95 (95% CI 10.74-11.16). In high-demand, high-intensity scenarios, efficient monitoring required a mean of 101.80 (95% CI 101.65-101.96) minutes and mean cost of US $56.92 (95% CI 56.84-57.01), while inefficient monitoring increased time to a mean of 146.32 (95% CI 145.92-146.71) minutes and mean cost of US $81.82 (95% CI 81.60-82.04).

**Conclusions:**

A stepped care approach can efficiently sustain engagement in DHIs by targeting support to higher-need participants. These findings offer actionable guidance for designing scalable, cost-effective interventions for real-world settings, as resource-efficient engagement strategies remain a persistent challenge for DHIs.

## Introduction

Digital health interventions (DHIs) offer a promising approach to support patient self-management and promote health behavior change [1,2] while addressing barriers related to availability, accessibility, geography, and time constraints [3-5]. Despite this potential, systematic reviews report mixed evidence on DHI effectiveness, with limited user engagement identified as a key barrier [6,7]. Because engagement is closely linked to intervention effectiveness [8-10], low and inconsistent engagement poses a major barrier to the scalability and sustainability of DHIs, especially in underserved rural populations where resources are constrained and few evidence-based strategies exist [11,12].

Human-supported strategies, such as personalized support, tailored feedback, and reminders, are consistently linked to better DHI engagement [12,13]. Yet, these strategies are often resource-intensive and costly, posing major challenges for scalability and implementation [12]. Consequently, there is a critical need to identify and evaluate scalable, resource-efficient strategies that can strengthen user engagement in DHIs.

A stepped care framework may offer a potential solution to this challenge. Stepped care models are designed to maximize resource efficiency by initiating treatment with low-intensity interventions and escalating to high-intensity options only as necessary [14-17]. Traditionally used in behavioral and mental health contexts [18,19], stepped care has been shown to improve access [20], reduce treatment duration [21,22], and lower costs [23,24]. In dietary interventions, stepped care has been applied in contexts such as eating disorders [25] and weight management [26]. However, its application specifically as an engagement strategy within DHIs remains unexplored.

To evaluate the feasibility and potential scalability of a stepped care engagement approach within DHIs, it is essential to understand its demand, impact on engagement, and implementation costs. However, current research offers limited evidence on these Core indicators, leaving a critical gap in how engagement strategies are operationalized within digital platforms. In particular, assessing stepped care implementation costs from the provider’s perspective is particularly important when the long-term goal is to sustain implementation of DHIs within health care systems. Simulation methods enable systematic examination of how realistic variations in health care delivery (eg, shifts in demand for stepped care, monitoring efficiency, and stepped care intensity) influence total implementation costs and resource allocation [27]. By linking the engagement process with economic and operational dynamics, this framework provides a scalable, data-driven approach for optimizing DHIs and guiding implementation strategies in real-world settings.

This study presents secondary aims and analyses of iSIPsmarter, the experimental arm of a previously conducted randomized controlled trial (RCT). iSIPsmarter integrated a stepped care engagement framework, which is the focus of the present analysis. iSIPsmarter is a DHI aimed at reducing sugar-sweetened beverage (SSB) consumption among rural adults in Appalachia [28]. In an RCT, iSIPsmarter demonstrated effectiveness in reducing SSB intake following a 9-week intervention period (effect size=0.37; *P*=.005) and 6 months later (effect size=0.35; *P*=.009), as well as in improving percent weight loss at the 6-month assessment (effect size=0.23; *P*=.046), compared to a static patient education control group [29]. Engagement with the iSIPsmarter program was high, with most participants completing the majority of the educational modules (“Cores”) [29]. This high engagement, in part, might be attributed to the human-supported stepped care component designed to reengage disengaged participants. However, the demand and implementation cost of iSIPsmarter’s stepped care approach have not yet been examined and are the focus of this study. Furthermore, because these estimates are specific to the research context of the RCT, simulating how variations in demand and monitoring efficiency might affect implementation costs in real-world health care systems represents an essential step toward advancing iSIPsmarter along the translation pipeline.

Thus, the objectives of this study are 2-fold. First, this process evaluation addresses the trial’s a priori secondary aims by examining demand and implementation costs associated with a stepped care engagement approach [29,30]. Second, simulation analyses were conducted to explore additional research questions that were not an explicit focus of the original study. Specifically, the study simulates how variations in monitoring costs, demand, and stepped care intensity influence overall resource use and implementation costs to inform future implementation efforts.

## Methods

### Study Design

This study uses process data from a 2-group RCT where participants were randomized into either the iSIPsmarter experimental condition or the Patient Education website control, with both groups receiving content focused on reducing SSB [28]. Details on the effectiveness of the RCT and CONSORT (Consolidated Standards of Reporting Trials) diagrams can be found in previously published work [29]. The control group did not receive stepped care. Therefore, this study focuses solely on the stepped care data from the iSIPsmarter experimental condition, assessing both the demand for stepped care and the associated implementation cost. All costs reported are in US dollars (US $).

### Study Sample

The broader 2-group RCT recruited participants from Southwest Virginia and neighboring Appalachian counties. Eligibility criteria included being 18 years or older, English-speaking, and reporting the consumption of more than 200 calories per day from SSBs. A total of 249 participants consented, enrolled, and were randomized with 127 assigned to the iSIPsmarter intervention.

### iSIPsmarter Intervention Description

The iSIPsmarter intervention is based on the theory of planned behavior, incorporating behavior change strategies and health literacy techniques [28]. The development process of the iSIPsmarter intervention was further guided by the Internet Intervention Model [31], which emphasizes the role of user characteristics, environment, intervention content, level of support, and targeted outcomes in shaping behavior change [32], as well as practices from human-centered design and instructional design [32]. Additional details on the development process can be found in the iSIPsmarter development manuscript [32].

The iSIPsmarter intervention includes 6 interactive Cores delivering behavioral change and educational content through integrated text, audio, graphics, animation, and video. Each Core is enhanced by vignettes depicting relatable storylines and situations focused on goal setting, behavior change, overcoming barriers, and managing relapses. iSIPsmarter also included behavioral diary tracking and personalized action planning [28]. The only nondigital component is a human-supported stepped care strategy to encourage Core completion. As further described below, all iSIPsmarter participants have access to stepped care, although not all require it.

### Stepped Care Theoretical Rationale and Description

iSIPsmarter’s stepped care approach was informed by the Internet Intervention Model [31], specifically the domain addressing level of support, and by the Supportive Accountability Model, which posits that individuals are more likely to adhere to a DHI when they feel accountable to a supportive, trustworthy person who provides oversight and encouragement [12,13,33]. It was designed to promote reengagement among disengaged participants and to enhance behavioral engagement [34,35], specifically Core (module) completion. Unlike traditional stepped care approaches, which typically escalate intervention intensity through personalized coaching targeting behavioral outcomes [18-22,25] (eg, reductions in SSB intake), the iSIPsmarter stepped care approach focused on providing supportive accountability to encourage Core completion and address DHI disengagement [12,13]. This novel design may offer efficiency and scalability if future implementation efforts, particularly in settings where nutrition and weight management professionals are limited, such as low-resourced health care and community-based settings.

**Figure 1:**
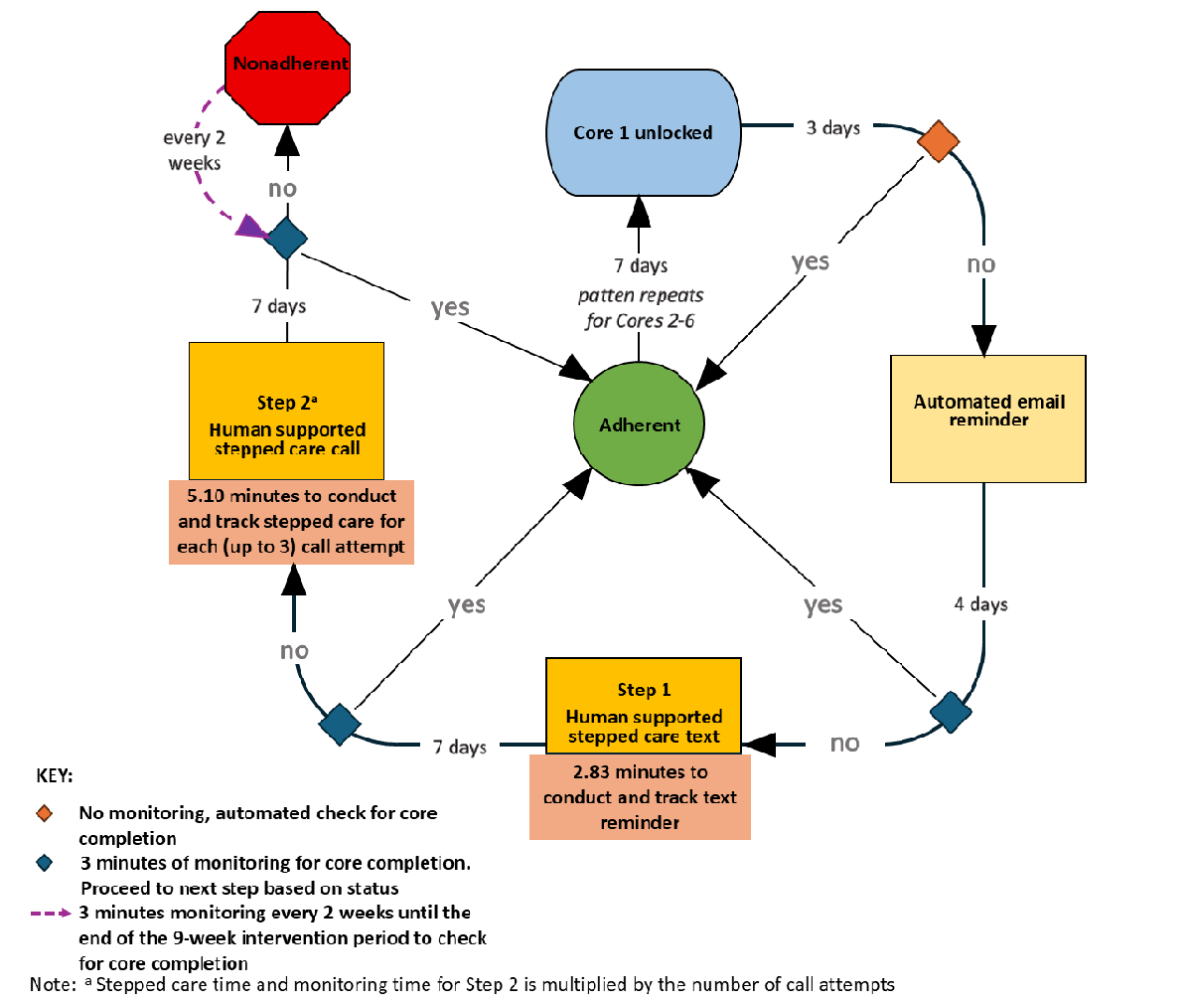
Stepped care approach to promote Core (module) completion in the digital iSIPsmarter intervention for Appalachian adults.

iSIPsmarter’s stepped care process is illustrated in Figure 1. During the 9-week intervention period, each Core was unlocked 7 days after the prior Core was completed. Participants who did not complete a Core within 3 days of its availability received an automated email reminder. One week after the Core became available, a study coordinator assessed completion status, triggering an initial monitoring cost. If the Core remained incomplete, the research coordinator evaluated the need for human-supported stepped care and initiated step 1, which involved sending a text reminder. This step generated both a text cost and a monitoring cost to track engagement. If the Core was still incomplete after an additional 7 days, step 2 was initiated: a phone call was made (up to 3 attempts to reach the participant with voicemail if necessary), incurring a phone call cost. These calls provided encouragement, technical assistance, and strategies to support task completion. Subsequent follow-up generated additional monitoring and phone costs.

Participants who did not complete the Core within 7 days after the last phone call attempt were classified as nonadherent. Nonadherent participants included those reached by phone but who did not complete the Core as well as those who were never reached (despite leaving voicemails). Participants who spoke with study coordinators once did not receive additional calls for the same Core. These individuals continued receiving automated emails every 2 weeks but no further human-supported stepped care. However, if they completed the Core at any point during the 9-week intervention period, they regained adherence status, and the next Core was unlocked after 7 days. Participants who completed a Core after the 9-week intervention period were still able to access subsequent Cores, but their engagement data were no longer tracked. Throughout the intervention, nonadherent participants were monitored every 2 weeks, contributing to nonadherence monitoring costs. As participants were required to complete each Core before advancing to the next, some were unable to complete all Cores within the 9 weeks. The monitoring and engagement steps are detailed in Figure 1 and the time spent by study coordinators and costs incurred are defined in the subsection below.

### Definition of Time Spent and Costs Incurred in the Stepped Care Engagement Process

Each level the of stepped care engagement process incurs specific costs, primarily reflecting staff time required for monitoring and outreach. These costs and corresponding times are defined as follows:

1. Initial monitoring cost (CIM) or time (TIM): The cost or time of assessing Core completion 1 week after being available, following the automated email reminder.
2. Text cost (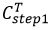) or time (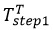): The cost or time of sending a text reminder (step 1) to noncompleters.
3. Monitoring text cost (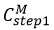) or time (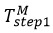): The cost or time of monitoring participant engagement after the text reminder.
4. Phone call cost (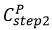) or time (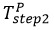): The cost or time of making up to 3 phone call attempts (step 2), including voicemails and participant support.
5. Monitoring phone cost (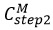) or time (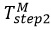): The cost or time of monitoring engagement following phone call attempts to determine the need for additional follow-up.
6. Nonadherence monitoring cost (CNAM) or time (TNAM): The cost or time of biweekly monitoring participants classified as nonadherent until the end of the intervention.

### Data Analysis

#### Stepped Care Engagement Demand

Engagement was measured by whether participants completed a Core, including following 1 of the 2 stepped care engagement strategies: the text reminder (step 1), or phone calls (step 2). Demand for stepped care was evaluated based on the extent to which the stepped care process successfully reengaged participants in the iSIPsmarter experimental condition. This approach provided a way to evaluate the success of each stepped care component in sustaining participant involvement and promoting Core completion. In addition to examining overall Core completion and aggregate stepped care demand, participant-level stepped care demand was tracked longitudinally to explore individual variation in support needs.

#### Stepped Care Implementation Time and Costs

Recent work on cost assessment for behavioral interventions highlights the importance of tracking staff time as a key driver of implementation costs [36-39]. Staff time, a major and variable cost, particularly for training and support, is often harder to estimate than material costs. Time-driven activity-based costing (TDABC) offers a practical assessment method [39-41]. In this study, implementation costs were assessed as the cost of delivering the stepped care engagement strategies in the iSIPsmarter experimental condition, using TDABC’s uniform estimate of time for tasks self-report approach [42].

For each participant, the average time and cost of implementing the stepped care process for each participant were calculated based on study coordinator-reported time data. Each of the 5 study coordinators (DJB, TM, AR, BK, and HW) provided time estimates for each step of the process across 3 separate instances. The average of these self-reported time estimates was included in the analysis with the monitoring time (defined as checking for Core completion) at 3 minutes, text time at 2.83 minutes, and phone call time at 5.1 minutes. The labor costs of stepped care were valued at the market wage rate for the associated occupation by taking the 2023 national mean wage for a “Health Education Specialist” reported as US $33.55/hour in the Occupational Employment and Wage Statistics (OEWS) data from the US Bureau of Labor Statistics [43]. The time estimates in minutes were converted to hours and multiplied with the mean wage to get a cost estimate of the time spent on each step of the stepped care process. For each of the 6 Cores, the per-participant cost of implementing the stepped care process was estimated based on the defined cost components. Initial monitoring (US $1.68) covered checking the Core completion after the automated email. Text (US $1.58) covered typing and sending the text and monitoring text (US $1.68) accounted for tracking completion after the text reminder (step 1). Phone (US $2.85) covered up to 3 call attempts, while monitoring phone (US $1.68) tracked completion after each attempt. Nonadherence monitoring (US $1.68) reflected biweekly checks for participants who remained nonadherent.

### Simulation Models

The iSIPsmarter trial was conducted with a relatively small and homogeneous sample, and the observed demand and engagement patterns are specific to a controlled research context. To explore how these dynamics might vary in applied settings, we used deterministic scenario simulations [44] to assess how anticipated variations in demand, monitoring costs, and stepped care intensity influence overall resource use and implementation costs.

These simulations are not intended to test the robustness of a single empirical estimate, as would be the case in a traditional sensitivity analysis. Instead, they operationalize a scenario-driven framework that links engagement dynamics with implementation costs to anticipate how a stepped care engagement strategy would perform under diverse real-world conditions, which is the information that cannot be obtained from the trial alone. Specifically, the simulation parameters reflect resource allocation frameworks, highlighting the need to balance intervention effectiveness with cost efficiency [44]. A total of 18 distinct scenarios were modeled to explore these variations. Because the 6 Cores were unlocked sequentially based on prior completion, the simulations use longitudinal nested data draws to maintain the logical flow of Core access throughout the intervention.

Monitoring costs were systematically varied by reducing them to 20%, 50%, and 80% of the trial-observed per-participant stepped care implementation costs related to monitoring, reflecting decreasing monitoring efficiency encompassing all monitoring-related components: initial monitoring, text reminders, phone outreach, and nonadherence monitoring. These levels illustrate a plausible range of monitoring intensity reductions likely in real-world implementation.

Additionally, demand for stepped care was also varied at 3 levels (ie, 20%, 50%, and 80% of participants) to represent different degrees of need for human-supported engagement during the intervention. These scenarios were selected to provide a wide range of plausible conditions, informed by the observed heterogeneity in engagement with iSIPsmarter [45] as well as prior literature documenting variability in participant adherence and engagement with DHIs [46,47].

The intensity of stepped care was categorized into two levels: (1) low intensity: participants required only the initial monitoring step of automated email and text reminders to reengage (step 1); and (2) high intensity: participants required the full stepped care engagement process, including up to 3 phone calls (step 2) to reengage them. This reflected the study design and illustrated cost differences between lower- and higher-intensity support.

The total simulated cost *C_total,i_* for each scenario was modeled as a function of the monitoring costs, the demand for stepped care, and the intensity of the stepped care process, as shown in the equations below.

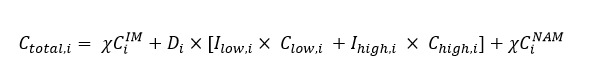

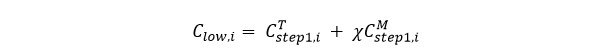

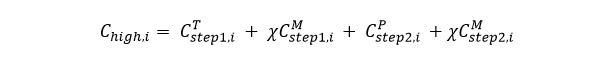







where *i* represent individual participant; χ ∈{0.2,0.5,0.8} is the monitoring efficiency multiplier, applied to all monitoring-related costs; 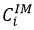 is the cost of initial monitoring; *D_i_* ∈{0,1} indicates whether participant *i* receives stepped care (1 if required, 0 otherwise), with stepped care requirements governed by the demand scenario parameter δ ∈{0.2,0.5,0.8}, which represent the proportion of participants receiving stepped care in each simulation scenario; *I_low_* ∈{0,1} is an indicator equal to 1 if the participant receives only step 1 (ie, text-based engagement); *I_high_* ∈{0,1}is an indicator equal to 1 if the participant receives step 1 and step 2 (ie, phone-based engagement); *C_low_* is the cost of participants receiving only step 1; *C_high_* is the cost of participants receiving step 1 and step 2; 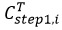 is the cost of tracking and sending text reminders; 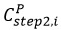 is the cost of conducting phone call attempts and/or voicemail; 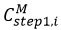 is the cost of monitoring response to text reminders; 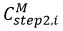 is the cost of monitoring response to phone calls; and 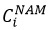 is the cost of monitoring for nonadherence after step 2. The indicators *I_low,i_* and *I_high,i_* are mutually exclusive (ie, *I_low,i_* + *I_high,i_* = 1) for participants who receive stepped care, reflecting that only one intensity level applies per participant.

By varying the demand for care and the intensity of the intervention, the model simulates the cost impact under different conditions of monitoring cost efficiency, participant engagement, and resource use. This simulation approach provides insights into how different components of the stepped care process contribute to the overall costs and resource needs, guiding optimization strategies for the intervention.

### Anticipated Simulation Patterns

We anticipated the following patterns to emerge from the simulations:

1. Among scenarios with the same percentage of stepped care demand, those with higher intensity engagement (eg, requiring phone calls) would result in higher overall implementation costs compared to those with lower intensity engagement (eg, text reminders only).
2. Among scenarios with the same stepped care demand and intensity level, those with more efficient monitoring (ie, lower monitoring cost) would result in lower overall implementation costs than those with less efficient monitoring (higher monitoring cost).

### Ethical Considerations

All study procedures were approved by the University of Virginia Institutional Review Board (#22130) and were registered prospectively at ClincialTrials.gov as NCT05030753 on August 26, 2021. There were no deviations from the trial registration. The study is described in accordance with the Template for Intervention Description and Replication (TIDieR) checklist (Multimedia Appendix 1) [48] to enhance transparency and reproducibility of reporting. We also follow the CONSORT-EHEALTH reporting guidelines for RCTs of eHealth interventions (Multimedia Appendix 2) [49]. Prior to enrollment in the RCT [28], participants provided written informed consent to participate in the study, including consent for the data presented in this paper. The paper and all supplementary materials do not include any photographs, screenshots, or other images that could permit identification of individual participants or users. Participants were allowed to withdraw from the study at any time. All data used in this analysis were deidentified to ensure participant privacy and confidentiality. Participants received compensation in the form of a cellular-enabled scale at baseline and gift cards (US $50) for completing each of the assessments.

## Results

### Demographics

**Figure 2:**
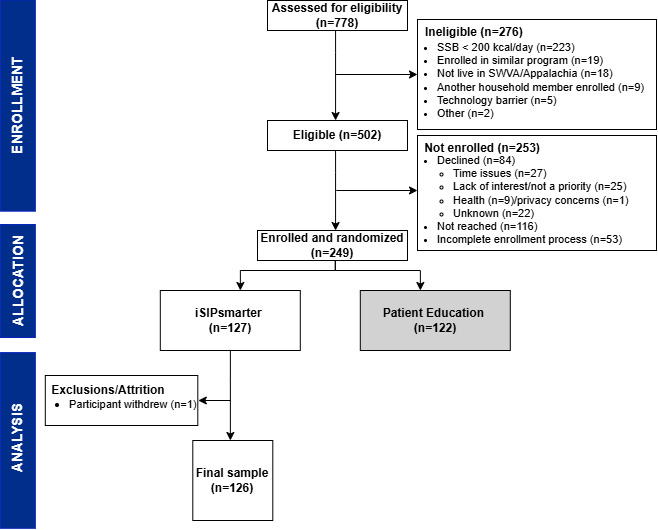
CONSORT (Consolidated Standards of Reporting Trials) diagram for a 2-arm randomized controlled trial of Appalachian adults, with analyses focused on stepped care within the iSIPsmarter arm. SSB: sugar-sweetened beverage; SWVA: southwest Virginia.

As shown in the CONSORT diagram (Figure 2), 778 individuals were assessed for eligibility, of which 502 (65%) were eligible and 249 (50%) were enrolled and randomized. Among the 127 participants in the iSIPsmarter intervention, 1 participant withdrew from the study midway and was consequently dropped from subsequent analysis. Analyzed participants (N=126) were predominantly women (n=102, 81%) and White (non-Hispanic) (n=112, 89%), with an average age of 41.9 (SD 12.01) years. Most participants (n=112, 89%) had at least some college education. Half of the participants (n=62, 50%) reported an annual income of US $55,000 or more. The mean health literacy score was 11.57 out of 12 (SD 0.99). The characteristics of the participants are given in Table S1 in Multimedia Appendix 3.

### Stepped Care Engagement Demand

Aggregated stepped care contact information showed that 76 (60%) participants required at least 1 stepped care contact over the course of the intervention, with participants averaging 1.2 stepped care contacts (Table S2 in Multimedia Appendix 3). On average, iSIPsmarter participants completed 5.2 (SD 1.6) of 6 total Cores, with 74 (59%) participants completing all 6 Cores and an additional 20 (16%) participants completing 5 Cores.

Table 1 summarizes participant demand for components of the stepped care process for each Core and the subsequent Core completion rates after each step of the stepped care process. As shown, the overall demand for text reminders (step 1) was approximately twice that of phone calls (step 2), with totals of 148 (range across Cores: 14-34) and 73 (range across Cores: 7-20), respectively. Among those who required stepped care, low-intensity text reminders (step 1) were generally effective: in 75 (51%) instances, participants successfully completed the Core after receiving the text reminders (Table 1). In contrast, high-intensity phone outreach (step 2) had a lower success rate, with 12 (16%) instances of recipients completing the Core after receiving the first phone call or voicemail, 6 (10%) instances after the second and 3 (5%) instances after the third (Table 1), leading to an overall success rate of 29% for step 2.

**Figure 3:**
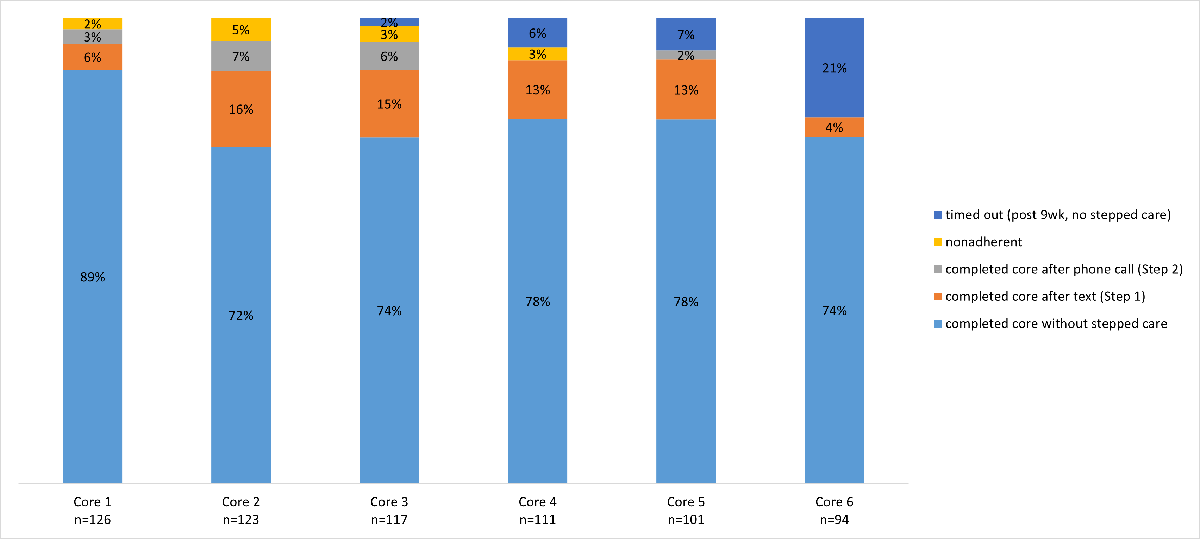
Aggregate participant completion rates for each Core (module) by stepped care levels in the digital iSIPsmarter intervention among Appalachian adults (data represent the initial 9-week intervention period).

Figure 3 illustrates the distribution of engagement with the stepped care levels for each Core. Across the 6 Cores, the majority of participants completed each Core without requiring stepped care, indicating that a substantial proportion of participants remained engaged with the iSIPsmarter intervention without requiring additional support. Step 1 and Step 2 showed varied success across the Cores, with Core completion after Step 1 and Step 2 peaking at Core 2. Core nonadherence after stepped care was low. Because participants could not proceed to the next Cores without completing the previous one, some did not finish all 6 Cores within the 9-week intervention period. By the end of the 9-week intervention period, 36 participants had timed out, including 20 who were still on Core 6.

**Table 1:** Demand and Core (module) completion success for each level of stepped care within the digital iSIPsmarter^a^ intervention among Appalachian adults.

Core	Needed step 1 (text)	Completed Core after step 1 (text)	Needed Step 2 (phone call)	Completed Core after phone call 1	Completed Core after phone call 2	Completed Core after phone call 3
Core 1	14	7	7	4	0	0
Core 2	34	20	14	6	0	2
Core 3	30	17	13	0	6	1
Core 4	24	14	10	0	0	0
Core 5	22	13	9	2	0	0
Core 6	24	4	20	0	0	0
Total^b^	148	75	73	12	6	3
Impact^c^	—^d^	51	—	16	10	5

^a^iSIPsmarter is a digital health intervention aimed at reducing sugar-sweetened beverage consumption; data represent the initial 9-week intervention period.

^b^Raw count of participants who needed each level of stepped care or completed Cores after each type of stepped care contacts, across all Cores.

^c^Impact of stepped care contacts given by the percentage of participants who completed Cores after receiving stepped care contacts (for text and phone calls).

^d^Not applicable.

Longitudinal tracking of participant-level stepped care demand revealed that support was used by many participants across Cores, rather than concentrated among a few. This pattern is illustrated in Figure S1 in Multimedia Appendix 3.

### Stepped Care Implementation Time and Costs

Table 2 presents descriptive statistics for the time and cost associated with each component of the stepped care process. On average, implementing the full stepped care process required 26.46 (SD 11.02) minutes per participant and cost US $14.80 (SD 6.16; Table 2). The initial monitoring step was the most resource-intensive, averaging US $8.51 (SD 2.35) and accounting for 58% of the total implementation cost. Altogether, monitoring-related costs, including initial, text, phone, and nonadherence monitoring, comprised US $11.61, or 78% of the total average cost (Table 2).

Implementation costs varied across Cores. Core 2 had the highest average cost (US $3.52, SD 4.2) and time commitment (6.3, SD 7.51 minutes), while Core 6 had the lowest average cost (US $1.17, SD 1.17) and time (2.09, SD 2.1 minutes; Table 2).

**Figure 4:**
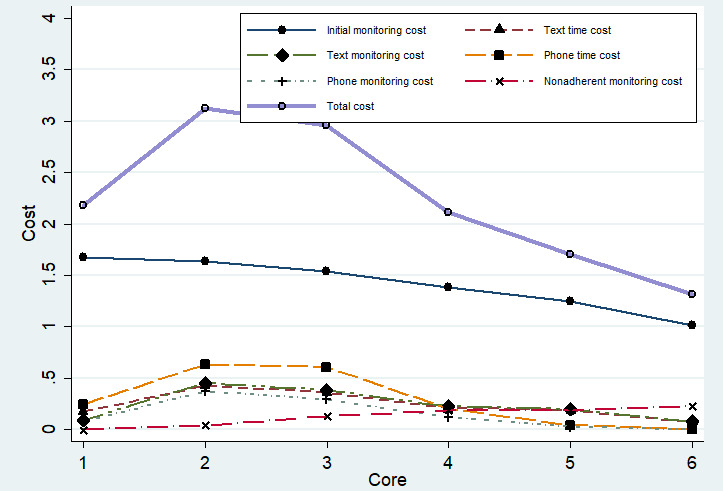
Distribution of stepped care engagement cost (in US $) across the 6 Cores (modules) of the digital iSIPsmarter intervention among Appalachian adults (data represents the initial 9-week intervention period).

Figure 4 illustrates the distribution of stepped care component costs by Core. Initial monitoring was the highest cost across all Cores, peaking at Core 1 and gradually declining. Costs for step 1 (text + text monitoring) and step 2 (phone + phone monitoring) peaked at Core 2, then declined in subsequent Cores. In contrast, nonadherence monitoring costs rose over time, peaking at Core 6. The total Core-specific cost, the sum of all components, was highest at Core 2 and declined thereafter.

**Table 2:** Descriptive statistics (time and cost) of stepped care implementation for the digital iSIPsmarter^a^ intervention among Appalachian adults.

Variable^b^			Time (min), mean (SD; range)		Cost (US $), mean (SD; range)
Total			26.46 (11.02; 15.00-58.16)		14.80 (6.16; 8.39-32.52)
Initial monitoring			15.21 (4.19; 3-18)		8.51 (2.35; 1.68-10.07)
Text (step 1)			2.58 (2.58; 0-8.49)		1.44 (1.44; 0-4.75)
Monitoring text (step 1)			2.57 (2.57; 0-9)		1.44 (1.43; 0-5.03)
Phone (step 2)			3.12 (5.88; 0-25.5)		1.74 (3.29; 0-14.26)
Monitoring phone (step 2)			1.60 (3.21; 0-12)		0.89 (1.80; 0-6.71)
Nonadherent monitoring			1.38 (3.79; 0-15)		0.77 (2.12; 0-8.39)
**By Core**					
	Core 1	4.07 (4.31; 3.00-33.13)		2.28 (2.41; 1.68-18.53)	
	Core 2	6.30 (7.51; 0-33.13)		3.52 (4.20; 0-18.53)	
	Core 3	5.72 (7.27; 0-33.13)		3.20 (4.06; 0-18.53)	
	Core 4	3.84 (5.14; 0-33.13)		2.15 (2.88; 0-18.53)	
	Core 5	3.06 (2.99; 0-16.93)		1.71 (1.67; 0-9.47)	
	Core 6	2.09 (2.10; 0-8.83)		1.17 (1.17; 0-4.94)	

^a^iSIPsmarter is a digital health intervention aimed at reducing sugar-sweetened beverage (SSB) consumption; data represent the initial 9-week intervention period

^b^The sample size for the stepped care intervention is 126.

### Simulations

Table 3 presents the aggregate cost estimates from the deterministic scenario simulation modeling, varying stepped care demand, monitoring efficiency, and stepped care intensity, with corresponding time estimates provided in Table S4 in Multimedia Appendix 3. Total simulated costs varied substantially across stepped care demand, monitoring cost efficiency, and stepped care intensity, ranging from mean cost of US $4.18 (95% CI 4.12-4.24) in the low demand, high monitoring efficiency, and low intensity scenario to mean cost of US $81.82 (95% CI 81.60-82.04) in the high demand, low monitoring efficiency, and high intensity scenario (Table 3). Similarly, time estimates varied across the different simulation scenarios with total time spent on low demand, high monitoring efficiency, and low intensity scenario estimated at mean time of 7.47 (95% CI 7.36-7.57) minutes while total time spent on high demand, low monitoring efficiency, and high intensity scenario was estimated at mean time of 146.32 (95% CI 145.92-146.71) minutes (Table S4 in Multimedia Appendix 3). Consistent with the anticipated simulation (see “pattern 1” in the section “Anticipated Simulation Pattern”), high-intensity stepped care resulted in substantially greater implementation costs than low-intensity care across all demand and monitoring levels. For example, under a high-demand scenario (101/126, 80%) of participants requiring stepped care), shifting from high- to low-intensity care decreased total costs by US $60.37 and time by 107.96 minutes, a 74% decrease, under inefficient monitoring, and by US $45.87 and 82.03 minutes, an 81% decrease, under efficient monitoring. This cost and time pattern was consistent across moderate (63/126, 50%) and low (25/126, 20%) demand scenarios. Likewise, consistent with anticipated simulation (see “pattern 2” in the section “Anticipated Simulation Pattern”), reductions in monitoring costs led to substantial savings. Under low-intensity care and low demand, reducing monitoring costs from 80% to 20% of baseline levels resulted in a US $6.77 (62%) reduction in total costs. Under high-intensity care and high demand, this reduction yielded a US $24.9 (30%) decrease in total costs.

**Table 3:** Aggregate participant cost of implementation of stepped care engagement of the digital iSIPsmarter^a^ intervention among Appalachian adults: results from simulating varying levels of demand for stepped care, monitoring, and intensity of stepped care (values are means of 1000 simulations; in US $; mean, 95% CI)^b^.

Monitoring level^c^	Demand for stepped care^d^	Intensity^e^	*C_total_*	*C^IM^*	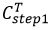	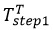	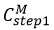	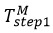	*C^NAM^*
Low	Low	Low	4.18; 4.12-4.24	1.70; 1.62-1.78	1.92; 1.90-1.94	0.40; 0.40-0.41	—^f^	—	0.15; 0.08-0.23
Low	Low	High	15.60; 15.51-15.70	1.70; 1.62-1.78	1.92; 1.90-1.94	0.40; 0.40-0.41	10.22; 10.15-10.29	1.21; 1.20-1.22	0.15; 0.08-0.23
Low	Medium	Low	7.61; 7.55-7.67	1.70; 1.62-1.78	4.75; 4.72-4.78	1.00; 0.99-1.01	—	—	0.15; 0.08-0.23
Low	Medium	High	36.28; 36.16-36.39	1.70; 1.62-1.78	4.75; 4.72-4.78	1.00; 0.99-1.01	25.64; 25.55-25.73	3.02; 3.01-3.03	0.15; 0.08-0.23
Low	High	Low	11.05; 10.99-11.11	1.70; 1.62-1.78	7.58; 7.56-7.61	1.61; 1.60-1.61	—	—	0.15; 0.08-0.23
Low	High	High	56.92; 56.84-57.01	1.70; 1.62-1.78	7.58; 7.56-7.61	1.61; 1.60-1.61	41.04; 40.98-41.11	4.83; 4.82-4.84	0.15; 0.08-0.23
Medium	Low	Low	7.56; 7.43-7.69	4.25; 4.05-4.46	1.92; 1.90-1.94	1.00; 0.99-1.01	—	—	0.39; 0.20-0.57
Medium	Low	High	20.80; 20.65-20.96	4.25; 4.05-4.46	1.92; 1.90-1.94	1.00; 0.99-1.01	10.22; 10.15-10.29	3.02; 3.01-3.04	0.39; 0.20-0.57
Medium	Medium	Low	11.90; 11.77-12.03	4.25; 4.05-4.46	4.75; 4.72-4.78	2.51; 2.50-2.52	—	—	0.39; 0.20-0.57
Medium	Medium	High	45.10; 44.93-45.27	4.25; 4.05-4.46	4.75; 4.72-4.78	2.51; 2.50-2.52	25.64; 25.55-25.73	7.56; 7.53-7.58	0.39; 0.20-0.57
Medium	High	Low	16.24; 16.12-16.38	4.25; 4.05-4.46	7.58; 7.56-7.61	4.02; 4.01-4.03	—	—	0.39; 0.20-0.57
Medium	High	High	69.37; 69.22-69.52	4.25; 4.05-4.46	7.58; 7.56-7.61	4.02; 4.01-4.03	41.04; 40.98-41.12	12.08; 12.06-12.10	0.39; 0.20-0.57
High	Low	Low	10.95; 10.74-11.16	6.81; 6.48-7.14	1.92; 1.90-1.94	1.61; 1.59-1.62	—	—	0.62; 0.32-0.92
High	Low	High	26.00; 25.78-26.23	6.81; 6.48-7.14	1.92; 1.90-1.94	1.61; 1.59-1.62	10.22; 10.15-10.29	4.84; 4.81-4.86	0.62; 0.32-0.92
High	Medium	Low	16.19; 15.99-16.40	6.81; 6.48-7.14	4.75; 4.72-4.78	4.02; 4.00-4.04	—	—	0.62; 0.32-0.92
High	Medium	High	53.92; 53.69-54.16	6.81; 6.48-7.14	4.75; 4.72-4.78	4.02; 4.00-4.04	25.64; 25.55-25.73	12.09; 12.05-12.13	0.62; 0.32-0.92
High	High	Low	21.44; 21.24-21.65	6.81; 6.48-7.14	7.58; 7.56-7.61	6.43; 6.42-6.45	—	—	0.62; 0.32-0.92
High	High	High	81.82; 81.60-82.04	6.81; 6.48-7.14	7.58; 7.56-7.61	6.43; 6.42-6.45	41.04; 40.98-41.11	19.33; 19.30-19.36	0.62; 0.32-0.92

^a^iSIPsmarter is a digital health intervention aimed at reducing sugar-sweetened beverage consumption; data represent the initial 9-week intervention period

^b^Refer to “Definition of Time Spent and Costs Incurred in the Stepped Care Engagement Process” for the definition of the cost variables.

^c^Monitoring levels: low (20% monitoring cost); medium (50% monitoring cost); high (80% monitoring cost).

^d^Demand for stepped care levels: low (20% participants needing stepped care); medium (50% participants needing stepped care); high (80% participants needing stepped care).

^e^Intensity levels: low (stepped care up to step 1); high (stepped care up to step 2).

^f^Not applicable.

Simulated cost estimates by each Core are presented in Table S3 in Multimedia Appendix 3 with the corresponding time estimates in Table S5 in Multimedia Appendix 3. For low demand, high monitoring efficiency, and low intensity scenario, Core 1 is estimated to have mean cost of US $0.72 (95% CI 0.72-0.73) and require mean of 1.29 (95% CI 1.29-1.30) minutes while Core 6 is estimated to mean cost of US $0.59 (95% CI 0.56-0.62) and require mean of 1.05 (95% CI 1.00-1.11) minutes (Tables S3 and S5 in Multimedia Appendix 3). Similar to aggregated results, time and cost estimates increased with high-resource intensity strategies, with higher demand for stepped care, and with low monitoring efficiency across all Cores.

**Figure 5:**
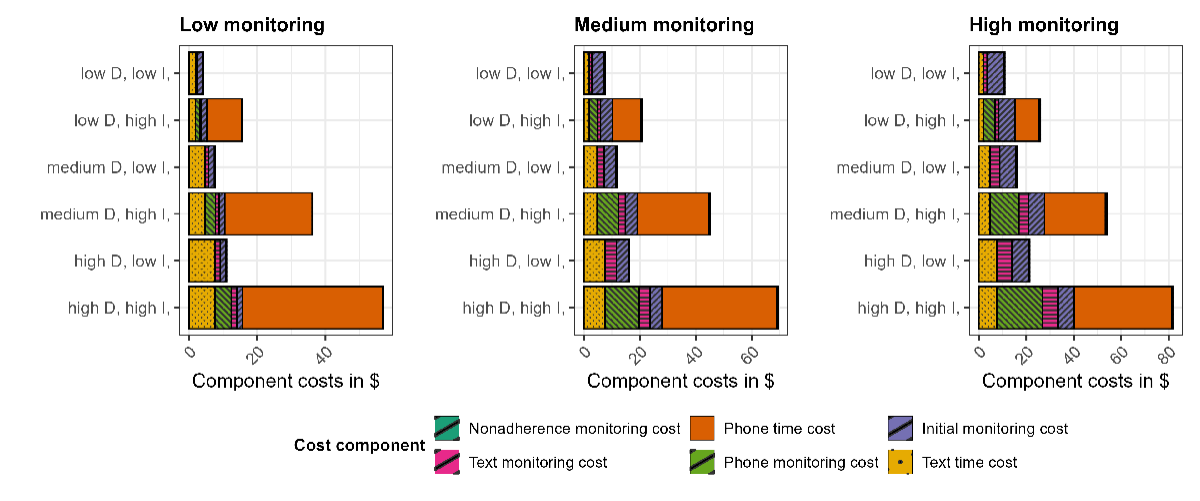
Distribution of the cost (in US $) of stepped care from simulating varying levels of demand for stepped care, monitoring, and intensity of stepped care for the digital iSIPsmarter intervention among Appalachian adults (data represent the initial 9-week intervention period).

Figure 5 visually compares the implementation costs across all simulation scenarios. Here, D denotes the demand for stepped care. I denotes intensity (low I - up to Step 1 [text reminders], high I - up to Step 2 [phone calls]). Each panel represents 1 of the 3 monitoring efficiency levels (low, medium, and high). Within each panel, cost components are stacked to illustrate their contribution under varying demand and intensity conditions. This figure highlights how total costs shift depending on simulation inputs and illustrates the dominant role of monitoring and intensity in driving cost differences across scenarios. For example, comparing the first panel (low monitoring) of high monitoring efficiency with the third panel (high monitoring) of low monitoring efficiency, the monitoring components (initial monitoring + text monitoring + phone monitoring + nonadherent monitoring) are noticeably larger in the third panel as compared to the first panel, illustrating the higher implementation cost of scenarios with inefficient monitoring. Similarly, comparing low- to high-intensity care, for higher intensity (high I) scenarios, costs of Step 2 components (phone time + phone monitoring) are added to the implementation costs as compared to lower intensity (low I) scenarios, under similar levels of monitoring and demand for stepped care. As also shown in Table 3, nonadherence monitoring contributes only a negligible share of total costs, which is why it appears nearly invisible in Figure 5.

## Discussion

### Principal Results

This study evaluated the demand and implementation costs of a stepped care engagement approach within the iSIPsmarter intervention and explored how variations in monitoring, demand, and intensity influence overall resource use. Our findings indicate that the intervention can engage most participants effectively without additional human support, while a subset of participants benefits from stepped care for specific or multiple intervention components. Monitoring was the largest driver of implementation costs, and simulation analyses demonstrated that both demand and intensity strongly influence total resource use. These results highlight the potential for adaptive, tiered engagement strategies to sustain participant involvement while efficiently allocating resources and providing actionable insights for optimizing future implementation of DHIs.

### Comparison With Prior Work

Our study introduces a novel application of stepped care to address one of the persistent challenges in DHIs, namely, sustaining user engagement [6,7]. Engagement in DHIs is a multifaceted construct, encompassing behavioral (ie, observable user actions within a DHI, such as Core completion), affective (ie, user’s emotional response, attitudes and feelings during a DHI), and cognitive (ie, mental effort, thought, and attention users invest during a DHI interaction) components [34,35]. Guided by the Supportive Accountability Model [33], which suggests that accountability to a supportive, trustworthy person enhances adherence, this study focused on a behavioral engagement strategy delivered by study personnel. While human-supported strategies, such as e-coaching (a technology-mediated process that provides guidance, feedback, and support to help individuals achieve behavior change), can enhance engagement [46], offering them universally is resource-intensive and often unsustainable. In contrast, a stepped care approach allocates resources efficiently, escalating human support only when disengagement occurs [14-17]. This is consistent with frameworks such as the Internet Intervention Model that emphasize the interplay of user needs, intervention features, and contextual factors in shaping engagement [12,13,31].

In our stepped care engagement approach, human-supported text messages and phone calls were used to reengage participants, troubleshoot barriers, and encourage completion of digital modules (Cores). Qualitative findings from a summative evaluation of iSIPsmarter indicated that participants valued these personal contacts, expressing appreciation for their interactions with the research team while complementary quantitative results showed that more than half of the participants believed that phone contact with an expert would have helped them be more successful [50]. Prior research on adding human facilitation into engagement strategies for DHIs has shown mixed results [51,52]. Although reviews suggest that incorporating human or social support can improve user experience and perceived engagement, effects on objective use outcomes are generally small and inconsistent across studies [52]. Remote facilitation delivered through telephone, email, or text-based coaching has shown particular promise in helping participants implement interventions and sustain adherence in a scalable and cost-efficient manner [12,13,53]. Consistent with this, findings from mobile health research suggest that hybrid interventions combining automated digital features with elements of human support tend to achieve higher adherence than unguided approaches [13].

Our findings suggest that while the iSIPsmarter intervention is relatively successful at engaging participants without additional human support, a subset of participants benefit from stepped care across specific or multiple Cores. This underscores the importance of flexible systems that can allocate intensive resources to participants with varying needs. Notably, support demand was distributed across many participants rather than concentrated among a few, suggesting that disengagement reflects a broad, recurring need rather than isolated cases. Although our analysis did not assess whether early high-intensity support predicted disengagement in later Cores, future research could build on these insights to identify patterns of sustained or escalating support needs to guide resource allocation. Such insights would help determine whether high resource intensity stepped care remains cost-efficient across all Cores or is most effective when limited to initial Cores. Future interventions could refine stepped care triggers by identifying participant characteristics linked to greater support needs, allowing for more personalized and targeted engagement strategies. This approach would be especially important for supporting participants who may face greater structural or social barriers to engagement. By tailoring resource allocation to those with higher needs, a stepped care engagement approach can help reduce disparities in DHI outcomes. Future research is needed to better understand how participant characteristics may also influence the demand for stepped care. However, our previous findings suggest that Core completion rates were not significantly affected by rurality, race, gender, income, or education [45]. Notably, older age was a significant predictor of higher Core completion. This suggests that while demand for stepped care may be relatively consistent across most demographic groups, younger participants may require more stepped care support to complete the Core modules. Our stepped care framework provides a generalizable framework for optimizing engagement in DHIs where sustained participation is essential. By optimizing resources, this novel design could support scalable implementation in health care and community settings with few providers. However, direct comparisons with other interventions are limited due to the unique design and context of this approach, emphasizing the need for further research to establish benchmarks and assess generalizability.

From a cost perspective, we used a TDABC approach to estimate the implementation cost of the stepped care engagement components [42]. Monitoring accounted for the majority of implementation expenses, highlighting it as a primary driver of resource use in our model of stepped care. Although the iSIPsmarter intervention was not designed to test the effectiveness of stepped care, it offers valuable insights into cost optimization strategies for stepped care engagement approaches. Simulation analysis demonstrated that demand and intensity strongly influence costs, with high-intensity, high-demand scenarios producing the greatest burden. Importantly, the simulated efficiency gains, reflecting potential automation of monitoring processes, indicate the potential for substantial cost savings across all demand levels. These findings point to a key opportunity for future DHI design: integrating scalable automation tools such as tracking systems, workflow platforms, or learning management systems, to reduce monitoring burden while preserving the human-supported elements critical to engagement. Prior literature on the application of simulation-driven decision support systems in health care highlights the value of using simulation to generate quantitative insights that inform effective resource management [54,55]. Such approaches have been applied to analyze capacity constraints, reduce costs, and identify optimal process configuration [56,57]. Thus, these simulation findings help define key parameters for progressing from the current RCT efficacy trial [29,32] to a hybrid effectiveness-implementation study in partnership with resource-constrained health care systems that may ultimately adopt stepped care engagement strategies within DHIs.

### Limitations

We acknowledge several limitations of this investigation. First, the estimates of demand and costs for the stepped care components are specific to the study population and intervention context, and as such, they cannot be readily generalized to other settings without further evidence. Second, the time data used in this study are self-reported and estimated by project staff, which may introduce response biases and affect the accuracy of the cost and time estimates. Third, the absence of a control group limits our ability to evaluate the effectiveness of stepped care in reducing SSB consumption or other behavioral outcomes. Fourth, we assume no change in Core completion rates in simulation scenarios with lower monitoring costs.

### Future Work

Future research should focus on exploring the effectiveness and cost-effectiveness of stepped care on both engagement and health outcomes, perhaps through an optimization trial whereby stepped care is experimentally varied (eg, turned on or off, or delivered at higher or lower intensity) [58]. Similarly, the integration of additional automated technologies should be explored to further enhance participant engagement and improve health outcomes, while containing implementation costs. Future research should also aim to optimize engagement by determining which types of support are most effective for reengaging participants and by identifying a priori which individuals are most likely to benefit from each approach. Finally, examining the success of stepped care engagement strategies across various digital health contexts and populations will help expand the generalizability of these findings.

### Conclusions

In conclusion, this study demonstrates an innovative application of stepped care to support engagement in DHIs, addressing a common challenge in sustaining user participation. Unlike most prior approaches that provide either universal human support [12,13] or fully automated interventions [59], this tiered, adaptive strategy selectively allocates support to participants who need it most. By showing how stepped care can maintain engagement efficiently while optimizing resource use, the findings contribute actionable insights for designing scalable and cost-effective interventions. This is especially timely and relevant to the field, particularly as DHIs rapidly expand while sustainable, resource-efficient engagement strategies remain a persistent challenge. In practice, these results suggest that integrating stepped care with automated technologies could enhance adherence, reduce disparities, and support sustainable implementation of digital health programs across diverse populations and resource-limited real-world settings.

## Appendix

Multimedia Appendix 1 TIDieR checklist.

Multimedia Appendix 2 CONSORT-eHEALTH checklist (V 1.6.1).

Multimedia Appendix 3 Tabular and visual summaries of all the analyses with the digital intervention.

## Abbreviations

CONSORT: Consolidated Standards of Reporting Trials
DHI: digital health intervention
OEWS: Occupational Employment and Wage Statistics
RCT: randomized controlled trial
SSB: sugar sweetened beverages
TDABC: time-driven activity-based costing
TIDieR: Template for Intervention Description and Replication

## Data Availability

The datasets generated or analyzed during this study are available from the corresponding author on reasonable request.
