# Supplementary material for: A Stepped Care Approach to Promoting Engagement in a Digital Health Intervention: Secondary Analysis of a Randomized Controlled Trial
Source: J Med Internet Res. 2026 May 4;28:e80921. doi: 10.2196/80921 (PMC13184598; doi:10.2196/80921)

**Multimedia Appendix 3**

**Table S1.** Summary statistics of participants (n=126) in the stepped-care engagement of the digital *i*SIPsmarter intervention among Appalachian adults.

| Characteristics |  |
| --- | --- |
|  |  |
| Age, mean(SD) | 41.87 (12.01) |
| Health literacy score, mean(SD) | 11.57 (0.99) |
| **Gender, n(%)** |  |
| Female | 102 (80.95%) |
| Male | 24 (19.05%) |
| **Race, n(%)** |  |
| Caucasian | 112 (88.89%) |
| African American | 9 (7.14%) |
| More than one race | 2 (1.59%) |
| Other | 3 (2.37%) |
| **Education, n(%)** |  |
| ≤High school graduate | 14 (11.11%) |
| Some college | 43 (34.13%) |
| College graduate | 29 (23.02%) |
| Graduate school | 40 (31.75%) |
| **Income, n(%)** |  |
| ≤US$14,999 | 9 (7.14%) |
| US$15,000-34,999 | 27 (21.43%) |
| US$35,000-54,999 | 26 (20.63%) |
| ≥US$55,000 | 62 (50.00%) |

**Table S2.** Summary of stepped care contacts for stepped care engagement of the digital *i*SIPsmarter intervention among Appalachian adults.

|  | **Mean (SD)** |
| --- | --- |
| Stepped care contacts needed | 1.20 (1.30) |
| At least 1 stepped care contact | 0.60 (0.49) |
|  |  |
| **Stepped care contacts by Core** | **# (%)** |
| 0 Cores | 52 (41.27%) |
| 1 Cores | 42 (33.33%) |
| 2 Cores | 26 (20.63%) |
| 3 Cores | 7 (5.56%) |

*i*SIPsmarter is digital health intervention aimed at reducing sugar-sweetened beverage (SSB) consumption; data represents the initial 9-week intervention period for the n=126.

**Table S3.** Aggregate cost of implementation of stepped care by individual Cores for stepped-care engagement of the digital *i*SIPsmarter intervention among Appalachian adults: results from simulating varying levels of demand for stepped care, monitoring, and intensity of stepped care (values are means of 1000 simulations) (in US$) [mean; [95% CI]]

| Monitoring level^a^ | Demand for stepped care^b^ | Intensity^c^ | Core 1 | Core 2 | Core 3 | Core 4 | Core 5 | Core 6 |
| --- | --- | --- | --- | --- | --- | --- | --- | --- |
|  |  |  |  |  |  |  |  |  |
| Low | Low | Low | 0.72; [0.72, 0.73] | 0.71; [0.70, 0.72] | 0.70; [0.68, 0.71] | 0.66; [0.64, 0.69] | 0.64; [0.61, 0.66] | 0.59; [0.56, 0.62] |
| Low | Low | High | 2.63; [2.61, 2.64] | 2.62; [2.60, 2.64] | 2.60; [2.58, 2.62] | 2.57; [2.54, 2.59] | 2.54; [2.51, 2.57] | 2.49; [2.46, 2.53] |
| Low | Medium | Low | 1.29; [1.29, 1.30] | 1.29; [1.28, 1.30] | 1.27; [1.25, 1.28] | 1.24; [1.21, 1.26] | 1.21; [1.18, 1.24] | 1.16; [1.13, 1.19] |
| Low | Medium | High | 6.07; [6.06, 6.09] | 6.06; [6.05, 6.08] | 6.05; [6.02, 6.07] | 6.01; [5.98, 6.04] | 5.99; [5.95, 6.02] | 5.94; [5.91, 5.97] |
| Low | High | Low | 1.87;  [1.86, 1.87] | 1.86; [1.85, 1.87] | 1.84; [1.82, 1.86] | 1.81; [1.79, 1.83] | 1.78; [1.76, 1.81] | 1.73; [1.71, 1.76] |
| Low | High | High | 9.51; [9.50, 9.52] | 9.51; [9.49, 9.52] | 9.49; [9.47, 9.51] | 9.46; [9.43, 9.48] | 9.43; [9.40, 9.46] | 9.38; [9.35, 9.41] |
| Medium | Low | Low | 1.33; [1.32, 1.33] | 1.31; [1.28, 1.33] | 1.26; [1.22, 1.30] | 1.18; [1.12, 1.24] | 1.11; [1.05, 1.18] | 0.99; [0.92, 1.07] |
| Medium | Low | High | 3.53; [3.52, 3.55] | 3.51; [3.49, 3.54] | 3.47; [3.42, 3.51] | 3.39; [3.33, 3.45] | 3.32; [3.25, 3.39] | 3.20; [3.12, 3.27] |
| Medium | Medium | Low | 2.05; [2.04, 2.05] | 2.03; [2.01, 2.05] | 1.98; [1.94, 2.02] | 1.90; [1.85, 1.96] | 1.84; [1.77, 1.90] | 1.72; [1.64, 1.79] |
| Medium | Medium | High | 7.58; [7.57, 7.60] | 7.56; [7.53, 7.59] | 7.52; [7.47, 7.56] | 7.44; [7.38, 7.50] | 7.37; [7.30, 7.44] | 7.25; [7.17, 7.32] |
| Medium | High | Low | 2.77; [2.77, 2.78] | 2.75; [2.73, 2.78] | 2.71; [2.67, 2.75] | 2.63; [2.57, 2.68] | 2.56; [2.50, 2.63] | 2.44; [2.37, 2.51] |
| Medium | High | High | 11.63; [11.62, 11.64] | 11.61; [11.58, 11.63] | 11.56; [11.52, 11.60] | 11.48; [11.42, 11.54] | 11.41; [11.35, 11.48] | 11.29; [11.22, 11.37] |
| High | Low | Low | 1.93; [1.92, 1.93] | 1.90; [1.86, 1.93] | 1.82; [1.76, 1.89] | 1.70; [1.61, 1.79] | 1.59; [1.49, 1.69] | 1.40; [1.28, 1.51] |
| High | Low | High | 4.44; [4.42, 4.45] | 4.41; [4.37, 4.45] | 4.33; [4.27, 4.40] | 4.20; [4.11, 4.30] | 4.10; [3.99, 4.20] | 3.91; [3.79, 4.02] |
| High | Medium | Low | 2.80; [2.80, 2.81] | 2.77; [2.73, 2.81] | 2.70; [2.63, 2.76] | 2.57; [2.48, 2.66] | 2.46; [2.36, 2.57] | 2.27; [2.15, 2.39] |
| High | Medium | High | 9.09; [9.08, 9.11] | 9.06; [9.02, 9.10] | 8.99; [8.92, 9.05] | 8.86; [8.76, 8.95] | 8.75; [8.64, 8.86] | 8.56; [8.44, 8.68] |
| High | High | Low | 3.68; [3.67, 3.68] | 3.65; [3.61, 3.68] | 3.57; [3.51, 3.64] | 3.44; [3.35, 3.53] | 3.34; [3.23, 3.44] | 3.15; [3.03, 3.26] |
| High | High | High | 13.74; [13.73, 13.75] | 13.71; [13.67, 13.75] | 13.63; [13.57, 13.70] | 13.51; [13.42, 13.60] | 13.40; [13.30, 12.50] | 13.21; [13.09, 13.33] |

^a^ Monitoring levels: low (20% monitoring cost); medium (50% monitoring cost); high (80% monitoring cost)

^b^ Demand for stepped care levels: low (20% participants needing stepped care); medium (50% participants needing stepped care); high (80% participants needing stepped care)

^c^ Intensity levels: low (stepped care up to Step 1); high (stepped care up to Step 2)

*i*SIPsmarter is digital health intervention aimed at reducing sugar-sweetened beverage (SSB) consumption; data represents the initial 9-week intervention period

**Table S4.** Aggregate participant time of implementation of stepped care engagement of the digital *i*SIPsmarter intervention among Appalachian adults: results from simulating varying levels of demand for stepped care, monitoring, and intensity of stepped care (values are means of 1000 simulations) (in minutes) [mean; [95% CI]]^a^

| Monitoring level^b^ | Demand for stepped care^c^ | Intensity^d^ | $T_{\mathrm{total}}$ | $T^{\mathrm{IM}}$ | $T_{step1}^{T}$ | $T_{step1}^{M}$ | $T_{step2}^{P}$ | $T_{step2}^{M}$ | $T^{\mathrm{NAM}}$ |
| --- | --- | --- | --- | --- | --- | --- | --- | --- | --- |
|  |  |  |  |  |  |  |  |  |  |
| Low | Low | Low | 7.47; [7.36, 7.57] | 3.04; [2.90, 3.19] | 3.43; [3.39, 3.48] | 0.72; [0.71, 0.73] | - | - | 0.28; [0.14, 0.41] |
| Low | Low | High | 27.90; [27.74, 28.07] | 3.04; [2.90, 3.19] | 3.43; [3.39, 3.48] | 0.72; [0.71, 0.73] | 18.27; [18.15, 18.40] | 2.16; [2.15, 2.17] | 0.28; [0.14, 0.41] |
| Low | Medium | Low | 13.61; [13.50, 13.72] | 3.04; [2.90, 3.19] | 8.49; [8.45, 8.54] | 1.80; [1.79, 1.81] | - | - | 0.28; [0.14, 0.41] |
| Low | Medium | High | 64.87; [64.67, 65.07] | 3.04; [2.90, 3.19] | 8.49; [8.45, 8.54] | 1.80; [1.79, 1.81] | 45.86; [45.70, 46.02] | 5.41; [5.39, 5.42] | 0.28; [0.14, 0.41] |
| Low | High | Low | 19.76; [19.66, 19.86] | 3.04; [2.90, 3.19] | 13.56; [13.52, 13.60] | 2.88; [2.87, 2.88] | - | - | 0.28; [0.14, 0.41] |
| Low | High | High | 101.80; [101.65, 101.96] | 3.04; [2.90, 3.19] | 13.56; [13.52, 13.60] | 2.88; [2.87, 2.88] | 73.40; [73.29, 73.52] | 8.64; [8.63, 8.66] | 0.28; [0.14, 0.41] |
| Medium | Low | Low | 13.52; [13.29, 13.76] | 7.61; [7.24, 7.98] | 3.43; [3.39, 3.48] | 1.79; [1.78, 1.81] | - | - | 0.69; [0.36, 1.02] |
| Medium | Low | High | 37.20; [36.93, 37.48] | 7.61; [7.24, 7.98] | 3.43; [3.39, 3.48] | 1.79; [1.78, 1.81] | 18.27; [18.15, 18.40] | 5.41; [5.38, 5.44] | 0.69; [0.36, 1.02] |
| Medium | Medium | Low | 21.28; [21.05, 21.52] | 7.61; [7.24, 7.98] | 8.49; [8.45, 8.54] | 4.49; [4.47, 4.52] | - | - | 0.69; [0.36, 1.02] |
| Medium | Medium | High | 80.65; [80.36, 80.95] | 7.61; [7.24, 7.98] | 8.49; [8.45, 8.54] | 4.49; [4.47, 4.52] | 45.86; [45.70, 46.02] | 13.51; [13.47, 13.56] | 0.69; [0.36, 1.02] |
| Medium | High | Low | 29.05; [28.82, 29.28] | 7.61; [7.24, 7.98] | 13.56; [13.52, 13.60] | 7.19; [7.17, 7.21] | - | - | 0.69; [0.36, 1.02] |
| Medium | High | High | 124.06; [123.79, 124.33] | 7.61; [7.24, 7.98] | 13.56; [13.52, 13.60] | 7.19; [7.17, 7.21] | 73.40; [73.29, 73.52] | 21.61; [21.57, 21.64] | 0.69; [0.36, 1.02] |
| High | Low | Low | 19.58; [19.21, 19.95] | 12.17; [11.58, 12.76] | 3.43; [3.39, 3.48] | 2.87; [2.84, 2.90] | - | - | 1.10; [0.57, 1.64] |
| High | Low | High | 46.50; [46.10, 46.90] | 12.17; [11.58, 12.76] | 3.43; [3.39, 3.48] | 2.87; [2.84, 2.90] | 18.27; [18.15, 18.40] | 8.65; [8.60, 8.70] | 1.10; [0.57, 1.64] |
| High | Medium | Low | 28.96; [28.59, 29.32] | 12.17; [11.58, 12.76] | 8.49; [8.45, 8.54] | 7.19; [7.15, 7.23] | - | - | 1.10; [0.57, 1.64] |
| High | Medium | High | 96.44; [96.02, 96.85] | 12.17; [11.58, 12.76] | 8.49; [8.45, 8.54] | 7.19; [7.15, 7.23] | 45.86; [45.70, 46.02] | 21.62; [21.55, 21.69] | 1.10; [0.57, 1.64] |
| High | High | Low | 38.35; [37.98, 38.71] | 12.17; [11.58, 12.76] | 13.56; [13.52, 13.60] | 11.51; [11.48, 11.54] | - | - | 1.10; [0.57, 1.64] |
| High | High | High | 146.32; [145.92, 146.71] | 12.17; [11.58, 12.76] | 13.56; [13.52, 13.60] | 11.51; [11.48, 11.54] | 73.40; [73.29, 73.52] | 34.57; [34.51, 34.63] | 1.10; [0.57, 1.64] |

^a^ Refer to section 1.4.1 for definition of the time variables

^b^ Monitoring levels: low (20% monitoring cost); medium (50% monitoring cost); high (80% monitoring cost)

^c^ Demand for stepped care levels: low (20% participants needing stepped care); medium (50% participants needing stepped care); high (80% participants needing stepped care)

^d^ Intensity levels: low (stepped care up to Step 1); high (stepped care up to Step 2)

*i*SIPsmarter is digital health intervention aimed at reducing sugar-sweetened beverage (SSB) consumption; data represents the initial 9-week intervention period

**Table S5.** Aggregate time of implementation of stepped care by individual Cores for stepped-care engagement of the digital *i*SIPsmarter intervention among Appalachian adults: results from simulating varying levels of demand for stepped care, monitoring, and intensity of stepped care (values are means of 1000 simulations) (in minutes) [mean; [95% CI]]

| Monitoring level^a^ | Demand for stepped care^b^ | Intensity^c^ | Core 1 | Core 2 | Core 3 | Core 4 | Core 5 | Core 6 |
| --- | --- | --- | --- | --- | --- | --- | --- | --- |
|  |  |  |  |  |  |  |  |  |
| Low | Low | Low | 1.29; [1.29, 1.30] | 1.28; [1.26, 1.30] | 1.24; [1.22, 1.27] | 1.19; [1.15, 1.23] | 1.14; [1.09, 1.19] | 1.05; [1.00, 1.11] |
| Low | Low | High | 4.70; [4.68, 4.72] | 4.68; [4.65, 4.71] | 4.65; [4.61, 4.69] | 4.59; [4.55, 4.64] | 4.55; [4.49, 4.60] | 4.46; [4.40, 4.52] |
| Low | Medium | Low | 2.32; [2.31, 2.32] | 2.30; [2.28, 2.32] | 2.27; [2.24, 2.30] | 2.21; [2.17, 2.25] | 2.16; [2.12, 2.21] | 2.08; [2.02, 2.13] |
| Low | Medium | High | 10.86; [10.83, 10.89] | 10.84; [10.81, 10.88] | 10.81; [10.77, 10.85] | 10.75; [10.70, 10.81] | 10.71; [10.65, 10.77] | 10.62; [10.56, 10.68] |
| Low | High | Low | 3.34; [3.33, 3.35] | 3.33; [3.31, 3.34] | 3.29; [3.26, 3.32] | 3.24; [3.19, 3.28] | 3.19; 3.14, 3.24] | 3.10; [3.05, 3.15] |
| Low | High | High | 17.01; [16.99, 17.03] | 17.00; [16.98, 17.02] | 16.97; [16.93, 17.00] | 16.91; [16.87, 16.95] | 16.86; [16.81, 16.91] | 16.78; [16.72, 16.83] |
| Medium | Low | Low | 2.37; [2.36, 2.38] | 2.34; [2.29, 2.38] | 2.25; [2.18, 2.32] | 2.11; [2.01, 2.21] | 1.99; [1.87, 2.11] | 1.78; [1.65, 1.91] |
| Medium | Low | High | 6.32; [6.29, 6.34] | 6.28; [6.23, 6.33] | 6.20; [6.12, 6.27] | 6.06; [5.95, 6.16] | 5.94; [5.82, 6.06] | 5.72; [5.59, 5.86] |
| Medium | Medium | Low | 3.66; [3.66, 3.67] | 3.63; [3.59, 3.67] | 3.55; [3.47, 3.62] | 3.40; [3.30, 3.50] | 3.28; [3.17, 3.40] | 3.07; [2.94, 3.20] |
| Medium | Medium | High | 13.56; [13.53, 13.59] | 13.52; [13.47, 13.57] | 13.44; [13.36, 13.52] | 13.30; [13.19, 13.41] | 13.18; [13.06, 13.30] | 12.96; [12.83, 13.10] |
| Medium | High | Low | 4.96; [4.95, 4.97] | 4.92; [4.88, 4.96] | 4.84; [4.77, 4.91] | 4.70; [4.60, 4.80] | 4.58; [4.46, 4.69] | 4.36; [4.23, 4.49] |
| Medium | High | High | 20.79; [20.77, 20.81] | 20.76; [20.71, 20.80] | 20.67; [20.60, 20.75] | 20.53; [20.43, 20.63] | 20.41; [20.30, 20.53] | 20.20; [20.07, 20.33] |
| High | Low | Low | 3.45; [3.44, 3.46] | 3.39; [3.33, 3.46] | 3.26; [3.15, 3.38] | 3.03; [2.87, 3.19] | 2.84; [2.66, 3.03] | 2.50; [2.29, 2.71] |
| High | Low | High | 7.94; [7.91, 7.96] | 7.88; [7.81, 7.95] | 7.75; [7.63, 7.87] | 7.52; [7.35, 7.68] | 7.33; [7.14, 7.52] | 6.99; [6.77, 7.20] |
| High | Medium | Low | 5.01; [5.00, 5.02] | 4.96; [4.89, 5.02] | 4.82; [4.71, 4.94] | 4.59; [4.43, 4.76] | 4.40; [4.22, 4.59] | 4.06; [3.85, 4.27] |
| High | Medium | High | 16.26; [16.23, 16.29] | 16.20; [16.13, 16.28] | 16.07; [15.95, 16.19] | 15.84; [15.67, 16.01] | 15.65; [15.46, 15.84] | 15.31; [15.10, 15.52] |
| High | High | Low | 6.58; [6.57, 6.59] | 6.52; [6.46, 6.59] | 6.39; [6.27, 6.50] | 6.16; [6.00, 6.32] | 5.97; [5.78, 6.15] | 5.63; [5.42, 5.83] |
| High | High | High | 24.57; [24.55, 24.60] | 24.52; [24.45, 24.58] | 24.38; [24.27, 24.50] | 24.15; [23.99, 24.32] | 23.96; [23.78, 24.15] | 23.62; [23.41, 23.83] |

^a^ Monitoring levels: low (20% monitoring cost); medium (50% monitoring cost); high (80% monitoring cost)

^b^ Demand for stepped care levels: low (20% participants needing stepped care); medium (50% participants needing stepped care); high (80% participants needing stepped care)

^c^ Intensity levels: low (stepped care up to Step 1); high (stepped care up to Step 2)

*i*SIPsmarter is digital health intervention aimed at reducing sugar-sweetened beverage (SSB) consumption; data represents the initial 9-week intervention period

**Figure S1.** Need for stepped care across all cores of the digital *i*SIPsmarter intervention among Appalachian adults (n = 75); participants counted if they required stepped care at least once) during the initial 9-week period.


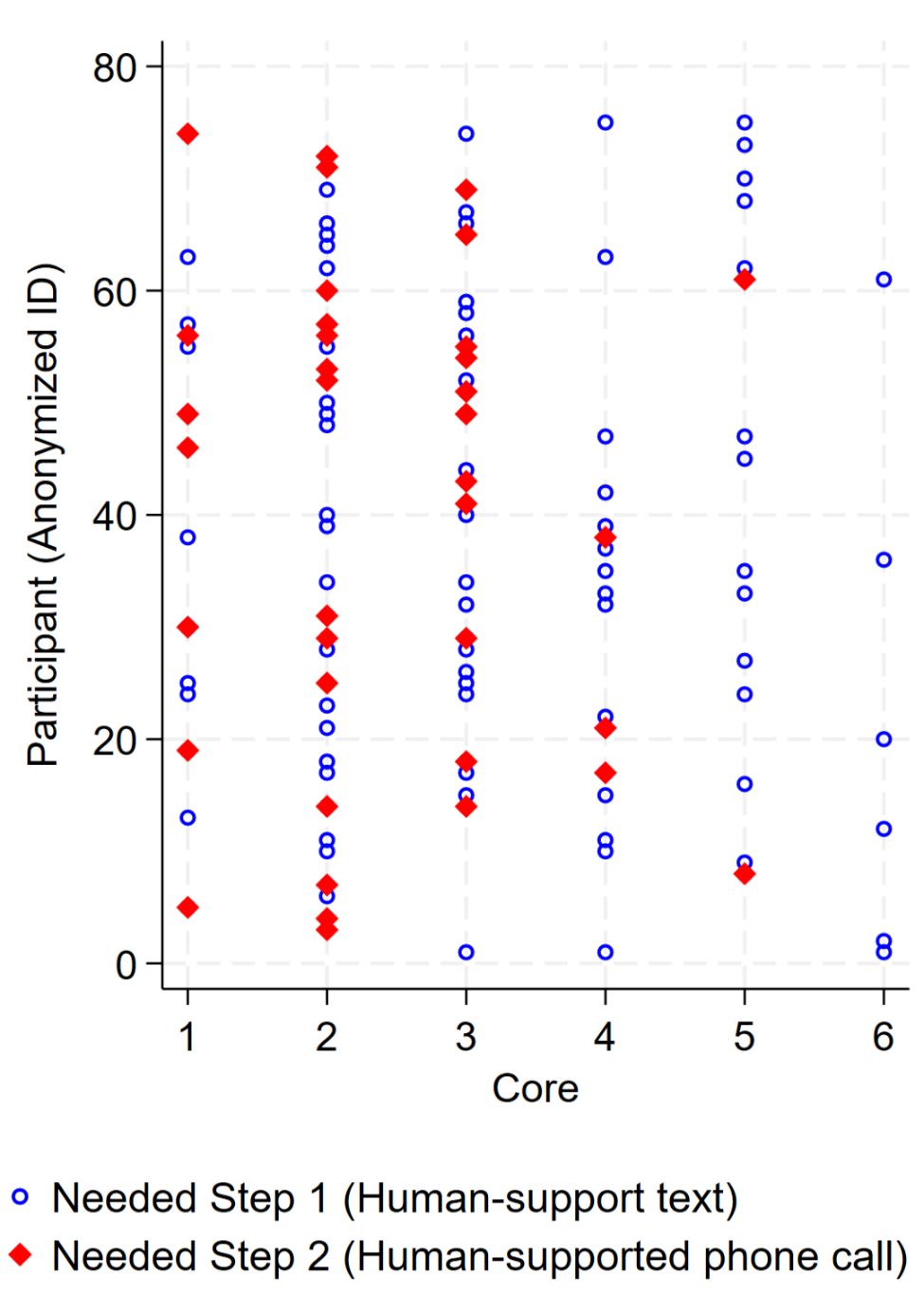

Supplement: Multimedia Appendix 3 [file jmir_v28i1e80921_app3.docx]
